# Sex differences between serum total bilirubin levels and cognition in patients with schizophrenia

**DOI:** 10.1186/s12888-021-03407-8

**Published:** 2021-08-10

**Authors:** Shen He, Yange Li, Tian Li, Feikang Xu, Duan Zeng, Yue Shi, Nan Zhao, Lei Zhang, Yin Zhu Ma, Qiang Wang, Wenjuan Yu, Yifeng Shen, Jingjing Huang, Huafang Li

**Affiliations:** 1 Department of Psychiatry, Shanghai Mental Health Center, Shanghai Jiao Tong University School of Medicine, Shanghai, China; 2 Department of Psychiatry, First Affiliated Hospital of Zhengzhou University, Zhengzhou, China; 3 Air Force Medical University, No. 169 Changle West Rd, Xi’an, 710032 China; 4 Shanghai Pudong New Area Mental Health Center, Tongji University School of Medicine, Shanghai, China; 5 Shanghai Clinical Research Center for Mental Health, Shanghai, China; 6 Shanghai Key Laboratory of Psychotic Disorders, Shanghai Mental Health Center, Shanghai Jiao Tong University School of Medicine, Shanghai, China

**Keywords:** Total bilirubin, Cognition, Schizophrenia

## Abstract

**Background:**

Cognitive deficits are common in patients with schizophrenia (SCZ). Abnormal serum total bilirubin (TBIL) levels have been involved in cognitive deficits associated with neuropsychiatric diseases such as mild cognitive impairment and subcortical ischemic vascular disease. However, this relationship has not yet been fully investigated in patients with SCZ. Therefore, the aim of this study was to investigate the association between the serum TBIL concentration and cognitive deficits in SCZ patients and to determine whether a sex difference exists in the association.

**Methods:**

A total of 455 participants were eligible and included in this cross-sectional study. Cognition was evaluated using the Montreal Cognitive Assessment. Serum TBIL concentration was measured with an automatic biochemistry analyzer according to the routine protocol in the hospital medical laboratory.

**Results:**

Serum TBIL levels were lower in the cognition impairment group than in the cognition normal group in male patients. In contrast, serum TBIL levels tended to be increased in the cognition impairment group in female patients, although the difference was not significant. Further stepwise multiple regression analysis stratified by sex showed that serum TBIL was independently and positively associated with cognitive function in male patients but not in female patients. Moreover, the association between serum TBIL level and cognitive function was also identified by the propensity score matching (PSM) method in male patients, but not in female patients.

**Conclusion:**

These findings suggest that lower serum TBIL levels may be associated with cognitive impairment in male SCZ patients.

## Background

Schizophrenia (SCZ) is a serious and potentially disabling mental disorder affecting approximately 0.5 to 1% of the general population worldwide [1]. While schizophrenia is known for a broad range of psychotic symptoms, research suggests that cognitive impairment is also frequent symptom occurring in patients with SCZ and has a greater impact on patient outcomes, social functioning and quality of life [2, 3]. Moreover, current antipsychotic medications do not adequately treat the persistent cognitive symptoms observed in SCZ [4, 5]. As such, it is crucial to determine the underlying neurobiological contributors to the pathophysiology of cognitive impairment in SCZ.

In recent years, oxidative stress has been suggested to contribute to the pathophysiology of SCZ [6–8]. Moreover, oxidative stress has detrimental effects on socio and neurocognitive abilities in SCZ, [9, 10] because redox dysregulation could impact structural and functional connectivity circuits, resulting in cognitive deficits [11, 12]. Martinez-Cengotitabengoa and colleagues were the first to explore the potential relationship between the levels of oxidative stress and neurocognition in schizophrenia, and found a significant correlation between glutathione (GSH) and executive function [13]. Furthermore, Cristina Gonzalez-Liencres et al. found that NT4/5, which has been shown to have antioxidant effects, appeared to have a potentially beneficial impact on neurocognition in SCZ [9].

Bilirubin, the end product of heme metabolism in the body, is an endogenous antioxidant with anti-inflammatory properties [14, 15]. Some studies have shown that bilirubin is associated with cognition. Serum concentrations of bilirubin were decreased in patients with mild cognitive impairment (MCI ) [16]. Moreover, some studies found a positive correlation between serum bilirubin levels and cognitive scores in patients with mild cognitive impairment and subcortical ischemic vascular disease [17, 18]. Based on the above research, we believe that serum TBIL concentration may influence cognitive function in SCZ patients, as oxidative stress is associated with cognitive function in SCZ and bilirubin has an antioxidant effect.

To date, there are very few studies investigating the relationship between serum TBIL concentration and cognition in SCZ patients. We found one recent study that reported an association between decreased serum TBIL concentration and immediate memory impairment in SCZ patients [19]. However, the sample size was small in their study. Moreover, some previous studies found that serum lipid levels may be associated with cognition in SCZ and that there is a close relationship between lipid metabolism and bilirubin [20–22]. Serum lipid levels as a potential important confounder were not considered in their study. Thus, it remains unknown whether serum TBIL is associated with cognition in SCZ patients independent of serum lipids.

On the other hand, although women and men with SCZ show similar neuropsychological damage^,^ [23] sex differences in cognition for SCZ have been well recognized [24–28]. Female SCZ patients have better functional outcomes and less cognitive impairment [25]. Zhang et al. found that male SCZ patients showed worse cognition than females in social cognition, processing speed, working memory, verbal learning and visual learning [25]. Other researchers had similar findings [26–28]. Moreover, sex differences in oxidative stress have been reported in numerous basic and clinical studies, wherein male SCZ patients exhibit higher oxidative stress than female SCZ patients [29, 30]. However, to date, the sex difference in the relationship between serum TBIL and cognition in patients with SCZ has not been investigated.

Therefore, in this paper, we wanted to examine (1) whether serum TBIL levels are independently associated with cognition in SCZ patients with a relatively large sample size and (2) whether sex differences exist between serum TBIL and cognitive impairment in SCZ patients.

## Methods

### Ethics statement

The research protocol and informed consent were approved by the Institutional Review Board of the Shanghai Mental Health Center, Shanghai Jiao Tong University School of Medicine. Written informed consent was obtained from all participants and their guardians.

### Participants

This was a cross-sectional study. The study population was a subsample of the Long-term Outcomes for Schizophrenia by Atypical Antipsychotic Treatment in China (SALT-C) study, [31] which is a multicenter, observational clinical study to evaluate the safety and efficacy of atypical antipsychotics in real-world conditions (clinicaltrials.gov identifier: NCT02640911). In total, 612 patients completed the cognitive function evaluation process during the baseline SALT-C study period between October 2016 and March 2019. Patients aged> 65 years, with no serum TBIL level test or history of hepatobiliary disease (such as hepatitis, pancreatitis and cholecystitis), cancer, cardiovascular disease, organic brain disease, dementia and mental retardation) were excluded. A final total of 455 patients were eligible and included in this study. Eligible patients were aged 18–65 years and diagnosed with SCZ based on the Diagnostic and Statistical Manual of Mental Disorders, Fourth Edition (DSM-IV) [32] by certificated psychiatrists; and able to take atypical antipsychotic medications.

### Clinical and neuropsychological assessment

Demographic and clinical characteristics, including age, sex, education level and total course of disease were obtained. All antipsychotic drugs were converted into chlorpromazine equivalents using published guidelines [33]. Education level was defined as the duration of formal education starting from the elementary school level in years. Height and weight and body mass index were obtained using standard measurements. The body mass index was calculated as an individual’s weight in kilograms divided by the square of height in meters. Hypertension was defined as a systolic blood pressure of ≥140 mmHg and/or a diastolic blood pressure of ≥90 mmHg and/or the current use of antihypertensive medication. Diagnosis of diabetes mellitus (DM) data was identified based on medical records and medical history. Cognitive function was assessed using the Montreal Cognitive Assessment (MoCA) [34] by a trained rater. MoCA has been shown to be a useful cognitive screening instrument for people with SCZ [35–37]. Global cognitive function was divided into two categories: cognitive impairment if the score was < 26, and normal if the score was ≥26 [38, 39]. The PANSS scale was used to assess the severity of SCZ psychopathology [40]. All psychiatrists received training pertaining to these scales.

### Serum measurement

Fasting venous blood from participants was collected to measure biochemical parameters. The concentrations of serum TBIL (normal range 5–21 μmol/L) levels, total cholesterol (TC,0.00–5.18 mmol/L), triglyceride (TG, normal range:0.00–1.7 mmol/L), low density lipoprotein (LDL, normal range: 0.00–3.12 mmol/L) and high density lipoprotein (HDL, normal range:1.04–1.66 mmol/L in male, 1.1–1.74 mmol/L in female) were measured by an automatic biochemistry analyzer according to routine protocols in the hospital medical laboratory.

### Statistical analysis

Clinical characteristics were compared between the cognitive impairment group and the cognitive normal group using independent-samples t-test and the Mann-Whitney U test for normally and not normally distributed continuous variables, respectively. The Kolmogorov-Smirnov test was used to test for normality. The chi-square test or exact chi-square test was used for dichotomous variables when appropriate.

Separate analyses were carried out for men and women, after which the significance of sex interaction effects was examined. To determine whether sex moderated the association between TBIL and cognition, we constructed a logistic regression model that included only main effects for sex and TBIL. We then added an interaction term for sex and TBIL and tested whether the *p*-value of the interaction term was significant. Moreover, we also constructed a full logistic regression model including sex, TBIL, interaction term and all potential confounding factors to test whether the *p*-value of the interaction term was significant in this model.

Univariate and multivariate binary logistic regression analyses were conducted separately by sex to determine the relationship between TBIL and cognitive function (normal or impairment). The potential confounders involved in this study included age (years), total disease duration (months), diabetes mellitus (yes/no), hypertension (yes/no), education (years), total PANSS score; TC, TG, LDL, HDL, BMI and drug exposure (chlorpromazine equivalent). The adjusted variates in the multivariate stepwise logistic regression models came from the potential confounding factors that were significant in the univariate logistic regression model (*p* < 0.1). The odds ratios (ORs) and corresponding 95% confidence intervals (CIs) were calculated. The Box-Tidwell test was conducted to identify the assumption for log-linearity in continuous variables. Multicollinearity was examined by collinearity diagnostic statistics. Variance inflation factor (VIF) > 4 or tolerance< 0.25 may indicate a concern for multicollinearity in multivariate regression models [41]. No more than 15% of the LDL and TC data were missing, and we used the missForest package to impute missing values in the statistical program R [42]. Sensitivity analyses were conducted excluding participants with serum TBIL > 21 μmol/L [43, 44] to minimize the possibility that some abnormal conditions (i.e., asymptomatic hyperbilirubinemia and Gilbert syndrome) could influence the results.

On the other hand, because the cognitive impairment group and cognitive normal group differed significantly across some baseline characteristics in both male patients and female patients, to enhance the robustness of the results and further confirm the association between serum TBIL and cognitive function, we conducted a secondary analysis using the propensity score matching (PSM) method to adjust for variables including age, total disease duration (months), BMI, diabetes mellitus (yes/no), hypertension (yes/no), education (years), total PANSS total scores, TC, TGs, LDL, HDL and chlorpromazine equivalent (mg) separately for sex. A one-to-one matching requirement via the nearest-neighbor matching algorithm was performed to select matched pairs of patients. This analysis was conducted by the MatchIt package in R [45]. Univariate and multivariate binary logistic regression analyses were conducted separately by sex after PSM analysis.

All analyses were performed by IBM SPSS Statistics (version 17.0, Chicago, USA) and the statistical program R 3.6.0. All data are presented as the mean (standard deviation). A two-sided *P* value < 0.05 was considered significant.

## Results

### Demographic characteristics and clinical features of participants

**Fig. 1:**
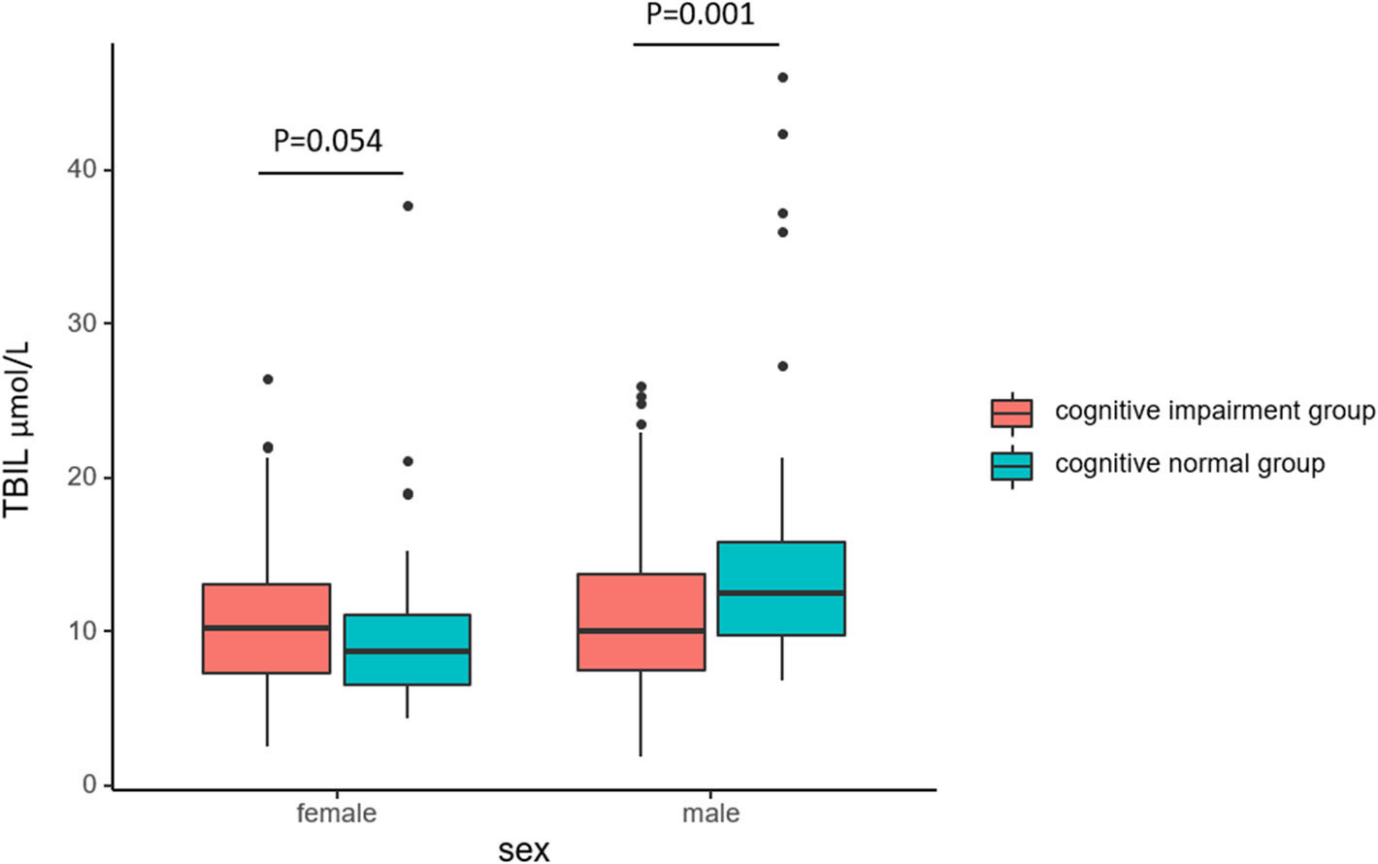
Comparison of serum TBIL levels between the cognitive impairment group and cognitive normal group according to sex

A total of 455 patients were included in this study. Of these patients, 367 patients were identified as the cognitive impairment group (mean MoCA score 18.02 ± 5.82). Eighty-eight patients were in the cognitively normal group (mean MoCA score 27.51 ± 1.18). The baseline characteristics of the study participants according to sex are summarized in Table 1. Compared with the cognitive normal group, the cognitive impairment group had older age, longer total disease duration and a shorter educational time in both male and female patients (all *p* < 0.001), as expected. Serum TBIL levels were lower in the cognitive impairment group than in the cognitive normal group for male patients (11.02 ± 4.77 μmol/L vs. 14.98 ± 8.75 μmol/L; *p* = 0.001). In contrast, serum TBIL levels tended to increase in the cognitive impairment group for female patients, although the difference was not significant (10.68 ± 4.22 μmol/L vs. 9.96 ± 6.04 μmol/L; *p* = 0.054) (Fig. 1).

**Table 1:** Demographic characteristics and clinical features of participants

	Male (*n* = 268)			Female (*n* = 187)		
	Cognition impairment group (*n* = 219)	Cognition normal group (*n* = 49)	*P* value	Cognition impairment group (*n* = 148)	Cognition normal group (*n* = 39)	*P* value
Age (years)	48.68 ± 11.71	37.10 ± 11.57	< 0.001*	47.84 ± 13.78	33.90 ± 10.97	< 0.001*
BMI (kg/m^2^)	24.57 ± 3.66	24.76 ± 3.92	0.749	24.25 ± 3.77	24.72 ± 4.34	0.53
Education (years)	9.70 ± 3.12	12.76 ± 3.06	< 0.001*	9.74 ± 3.86	13.21 ± 2.60	< 0.001*
Hypertension (yes/no)	30/189	0/49	0.002*	8/140	2/37	1.000
Diabetes mellitus (yes/no)	19/200	3/46	0.764	8/140	0/39	0.208
Total disease duration (months)	264.68 ± 142.16	157.12 ± 135.05	< 0.001*	232.57 ± 167.08	122.20 ± 111.92	< 0.001*
Total PANSS score	61.52 ± 17.54	61.00 ± 19.45	0.672	69.72 ± 18.91	55.31 ± 16.318	< 0.001*
TBIL (μmol/L)	11.02 ± 4.77	14.98 ± 8.75	0.001*	10.68 ± 4.22	9.96 ± 6.04	0.054
TC (mmol/L)	4.46 ± 0.90	4.59 ± 0.81	0.095	4.54 ± 0.89	4.68 ± 1.36	0.696
TG (mmol/L)	1.65 ± 1.24	1.74 ± 1.11	0.467	1.43 ± 0.92	2.08 ± 5.14	0.634
LDL (mmol/L)	2.82 ± 0.76	2.98 ± 0.84	0.169	2.74 ± 1.05	2.58 ± 0.75	0.351
HDL (mmol/L)	1.04 ± 0.26	1.29 ± 1.33	0.046*	1.32 ± 0.40	1.36 ± 0.27	0.220
Mean chlorpromazine equivalent (mg)	628.08 ± 379.8	578.97 ± 314.36	0.528	645.08 ± 378.02	614.42 ± 308.44	0.821

Values are presented as the mean ± SD. *p < 0.05. *PANSS* Positive and negative syndrome scale. *TBIL* total bilirubin. *TC* total cholesterol. *TG* triglyceride. *LDL* low density lipoprotein. *HDL* high density lipoprotein

### Association between serum TBIL level and cognition stratified by sex

Table 2 shows the results of the logistic regression analyses used to calculate odds ratios for the TBIL associated with cognition for men and women. For male patients, the unadjusted odds ratio (OR) between the cognitive normal group and cognitive impairment group was 1.079 (95%CI: 1.050–1.110; *p* < 0.001) for age, 0.719 (95%CI: 0.637–0.812; *p* < 0.001) for education, 1.006 (95%CI: 1.003–1.008; *p* < 0.001) for total disease duration, and 0.906 (95%CI:0.861–0.955; *p* < 0.001) for serum TBIL levels, respectively. For female patients, the unadjusted odds ratios between the cognition normal group and impairment group were 1.079 (95%CI: 1.047–1.112; *p* < 0.001) for age, 0.755 (95%CI: 0.671–0.851; *p* < 0.001) for education, 1.005 (95%CI: 1.002–1.008; *p* < 0.001) for total disease duration, and 1.053 (95%CI: 1.027–1.079; *p* < 0.001) for total PANSS score, respectively.

**Table 2:** Association Between Serum TBIL levels and cognitive function

	Male (*n* = 268)				Female (*n* = 187)			
	Univariate Results		Multivariate Results		Univariate Results		Multivariate Results	
	OR (95%CI)	*P* value	OR (95%CI)	*P* value	OR	*P* value	OR	*P* value
Age (years)	1.079 (1.050–1.110)	< 0.001*	1.064(1.032–1.098)	< 0.001*	1.079(1.047–1.112)	< 0.001*	1.061(1.026–1.098)	0.001*
BMI (kg/m^2^)	0.986 (0.908–1.072)	0.748	–	–	0.970(0.886–1.061	0.499	–	–
Education (years)	0.719 (0.637–0.812)	< 0.001*	0.729(0.640–0.831)	< 0.001*	0.755(0.671–0.851)	< 0.001*	0.831(0.728–0.950)	0.007*
Hypertension (yes/no)	NA	0.998	–	–	1.057(0.215–5.191)	0.945	–	–
Diabetes mellitus (yes/no)	1.457 (0.414–5.131)	0.558	–	–	NA	0.999	–	–
Total disease duration (months)	1.006 (1.003–1.008)	< 0.001*	NA	0.978	1.005(1.002–1.008)	< 0.001*	NA	0.311
Total PANSS score	1.002 (0.984–1.019)	0.853	–	–	1.053(1.027–1.079)	< 0.001*	1.042 (1.015–1.070)	0.002*
TBIL (μmol/L)	0.906 (0.861–0.955)	< 0.001*	0.931(0.873–0.992)	0.027*	1.037(0.954–1.127)	0.390	NA	0.447
TC (mmol/L)	0.847 (0.605–1.186)	0.335	–	–	0.876(0.624–1.228	0.442	–	–
TG (mmol/L)	0.944 (0.747–1.192)	0.626	–	–	0.922(0.802–1.059)	0.249	–	–
LDL (mmol/L)	0.781 (0.529–1.153)	0.213	–	–	1.217(0.782–1.896)	0.384	–	–
HDL (mmol/L)	0.418 (0.127–1.381)	0.153	–	–	0.756(0.302–1.894)	0.551	–	–
Mean chlorpromazine equivalent (mg)	1.000 (0.999–1.001)	0.399	–	–	1.000(0.999–1.001)	0.639	–	–

Dependent variable: cognitive function (0 = normal, 1 = impairment). *95%CI* 95% confidence interval. *OR* the odds ratio. *p < 0.05. PANSS=Positive and negative syndrome scale. *TBIL* total bilirubin. *TC* total cholesterol. *TG* triglyceride. *LDL* low density lipoprotein. *HDL* high density lipoprotein

These initial univariate analyses appeared to show similar patterns of odds ratios among men and women for most of the variables; however, the odds ratios for serum TBIL appeared to show a different trend. Thus, we examined interactions between sex and TBIL. We constructed a logistic regression model that included only main effects for sex and TBIL and then added an interaction term for sex and TBIL. Significant interaction effects were found between sex and TBIL (Wald statistic =7.263, *P* = .007). Subsequently, we entered all of the variables (including sex) into the model, together with the interaction effects of TBIL with sex. There was also a significant interaction (Wald statistic =4.661, *P* = .031). However, we did not observe a statistically significant interaction between TBIL and other factors, such as total disease duration, hypertension, diabetes mellitus, and drug exposure (Wald statistic =4.661, *P* = 0.387; Wald statistic =1.852, *P* = 0.174; Wald statistic =0.008, *P* = 0.930; Wald statistic =0.670, *P* = 0.413, respectively). Based on these results, it was necessary to perform a separate analysis for sex.

Further forward stepwise multiple logistic regression analysis with the forward LR method was performed to investigate the relationships between serum TBIL levels and cognition separated by sex. The association between TBIL and cognition in male patients remained statistically significant after adjustment for age, education, and total disease duration (OR = 0.931, 95% CI: 0.873–0.992, *p* = 0.027). However, the adjusted OR for TBIL was still not significant in female patients after adjustment for age, education, total disease duration, and PANSS total score (*p* = 0.447). Similar to univariate analysis, multivariate regression models showed a significant association between age and cognitive function (OR = 1.079, 95%CI:1.050–1.110, *p* < 0.001) in men and (OR = 1.061, 95% CI:1.047–1.112; *p* = 0.001).

women, as well as between education and cognitive function (OR = 0.729,95%CI: 0.640–0.831, *p* < 0.001) in men and (OR = 0.831; 95% CI: 0.728–0.950; *p* = 0.007) women. For sensitivity analysis, individuals (*n* = 25) who had serum TBIL concentrations > 21 μmol/L (1.2 mg/dL) were excluded. Multivariate regression models still showed a significant association between serum TBIL levels and cognitive function in men (OR = 0.899, 95% CI: 0.813–0.994, *p* = 0.038) but not in women (*p* = 0.132).

### Association between serum TBIL levels and cognitive function after PSM analysis

To enhance the robustness of the results, the propensity score-matching method was used, which resulted in balanced groups with underlying characteristics. Because our aim was to confirm the association between serum TBIL levels and cognition, we did not control for serum TBIL levels in the PSM analysis. After propensity matching, Table 3 shows that there were no differences between characteristics in cognition impairment group and cognition normal group for male and female patients (all p>0.05), except for total PANSS scores between two groups in female patients (*p* = 0.049). Moreover, serum TBIL.

**Table 3:** Demographics and clinical characteristics of propensity–matched patients

	Male (*n* = 98)			Female (*n* = 78)		
	Cognition impairment group (*n* = 49)	Cognition normal group (*n* = 49)	*P* value	Cognition impairment group (*n* = 39)	Cognition normal group (*n* = 39)	*P* value
Age (years)	38.43 ± 11.90	37.10 ± 11.57	0.513	35.79 ± 11.10	33.90 ± 10.97	0.415
BMI (kg/m^2^)	24.55 ± 3.96	24.76 ± 3.92	0.989	24.44 ± 3.23	24.72 ± 4.34	0.845
Education (years)	12.16 ± 3.23	12.76 ± 3.06	0.568	13.15 ± 3.65	13.21 ± 2.60	0.848
Hypertension (yes/no)	0/49	0/49	1	3/36	2/37	1
Diabetes mellitus (yes/no)	2/47	3/46	1	0/39	0/39	1
Total disease duration (months)	171.14 ± 110.28	157.12 ± 135.05	0.325	138.76 ± 135.27	122.20 ± 111.92	0.905
Total PANSS score	61.35 ± 20.11	61.00 ± 19.45	0.904	62.21 ± 17.77	55.31 ± 16.32	0.049*
TBIL (μmol/L)	11.05 ± 5.01	14.98 ± 8.75	0.03*	11.87 ± 4.72	9.96 ± 6.04	0.015*
TC (mmol/L)	4.47 ± 0.98	4.59 ± 0.81	0.134	4.57 ± 0.89	4.68 ± 1.36	0.916
TG (mmol/L)	1.80 ± 1.80	1.74 ± 1.11	0.654	1.52 ± 0.88	2.08 ± 5.14	0.325
LDL (mmol/L)	2.80 ± 0.81	2.98 ± 0.84	0.134	2.83 ± 1.65	2.58 ± 0.75	0.853
HDL (mmol/L)	1.09 ± 0.29	1.29 ± 1.33	0.601	1.38 ± 0.46	1.36 ± 0.27	0.503
Mean chlorpromazine equivalent (mg)	542.84 ± 350.92	578.97 ± 314.36	0.611	644.79 ± 320.76	614.42 ± 308.44	0.671

Values are presented as the mean ± SD. *p < 0.05. *PANSS* Positive and negative syndrome scale. *TBIL* total bilirubin. *TC* total cholesterol. *TG* triglyceride. *LDL* low density lipoprotein. *HDL* high density lipoprotein

levels were lower in the cognition impairment group than in the cognition normal group in males (11.05 ± 5.01 μmol/L vs. 14.98 ± 8.75 μmol/L; *p* = 0.03) but higher in the cognition impairment group than in the cognition normal group in female patients (11.87 ± 4.72 μmol/L vs. 9.96 ± 6.04 μmol/L; *p* = 0.015). Moreover, the forward stepwise multivariate regression results showed that there was a significant association between serum TBIL level and cognitive function (OR = 0.909, 95%CI: 0.841–0.983, *p* = 0.016) in men but not in women (*p* = 0.133) (Table 4), which was similar to the results before PSM analysis (Table 2).

**Table 4:** Association between serum TBIL levels and cognitive function after PSM analysis

	male (*n* = 98)				female (*n* = 78)			
	Univariate Results		Multivariate Results		Univariate Results		Multivariate Results	
	OR (95%CI)	*P* value	OR (95%CI)	*P* value	OR	*P* value	OR	*P* value
Age (years)	1.010(0.976–1.045)	0.573	–	–	1.015(0.976–1.056)	0.460	–	–
BMI (kg/m^2^)	0.986(0.891–1.092)	0.790	–	–	0.981(0872–1.103)	0.745	–	–
Education (years)	0.939(0.825–1.070)	0.345	–	–	0.995(0.863–1.072)	0.942	–	–
Hypertension (yes/no)	NA	1	–	–	1.542(0.243–9.776)	0.646	–	–
Diabetes mellitus (yes/no)	1.533(0.245–9.600)	0.648	–	–	NA	1.000	–	–
Total disease duration (months)	1.001(0.998–1.004)	0.571	–	–	1.001(0.997–1.005)	0.552	–	–
Total PANSS score	1.001(0.981–1.021)	0.930	–	–	1.025(0.996–1.055)	0.086	1.025(0.996–1.055)	0.086
TBIL (μmol/L)	0.909 (0.841–0.983)	0.016*	0.909 (0.841–0.983)	0.016*	1.074(0.979–1.178)	0.133	NA	0.133
TC (mmol/L)	0.852(0.543–1.337)	0.486	–	–	0.920(0.618–1.368)	0.679	–	–
TG (mmol/L)	1.030(0.786–1.348)	0.831	–	–	0.951(0.813–1.113)	0.534	–	–
LDL (mmol/L)	0.767(0.466–1.260)	0.295	–	–	1.191(0.786–1.805)	0.410	–	–
HDL (mmol/L)	0.701(0.279–1.759)	0.449	–	–	1.123 (0.336–3.752)	0.851	–	–
Mean chlorpromazine equivalent (mg)	1.000(0.998–1.001)	0.589			1.000(0.999–1.002)	0.667		

Dependent variable: cognitive function (0 = normal, 1 = impairment). *95% CI* 95% confidence interval. *OR* the odds ratio. *p < 0.05. *PANSS* Positive and negative syndrome scale. *TBIL* total bilirubin. *TC* total cholesterol. *TG* triglyceride. *LDL* low density lipoprotein. *HDL* high density lipoprotein

## Discussion

To the best of our knowledge, this is the first study that investigates sex differences in the relationship between serum TBIL levels and cognitive impairment in SCZ. Compared with cognitively normal patients, we found that the cognitive impairment group had lower serum TBIL levels in male patients but not in female patients. Moreover, serum TBIL levels were independently and positively associated with cognitive function in male patients but not in female patients.

To the best of our knowledge, only one previous study by Yin et al. investigated the relationship between serum TBIL and cognitive impairment in SCZ patients [19]. They reported that serum TBIL concentration was positively associated with the immediate memory score in SCZ patients. Their results support the hypothesis that TBIL plays a critical role in SCZ cognitive impairment. Our results were partially consistent with their finding. Our study also supported the hypothesis that defects in the antioxidant defense system might be involved in the cognitive impairment for SCZ patients. Moreover, our results showed that gender differences could exist.

A possible mechanism has been proposed to explain the effect of TBIL on cognition in SCZ patients. A decrease in NMDA receptor activity has been proposed as the basis for cognitive impairment in SCZ, [46, 47] and oxidative stress has been implicated in NMDA receptors hypofunction due to the redox-sensitive nature of these proteins [48, 49]. Moreover, it has been reported that neural bilirubin, as an endogenous antioxidant, can prevent NMDA receptor excitotoxicity by scavenging superoxide (O_2_^−^) [50]. As such, deficient levels of TBIL may contribute to NMDA hypofunction, resulting in cognitive decline. Our clinical findings suggested that a moderate increase in TBIL levels could be beneficial for cognition in male patients, not in female patients.

On the other hand, although the exact reasons for sex differences in the association between TBIL and cognition remain unclear, this difference may be explained by sex hormones. Increased oxidative stress can affect neuronal function and lead to impairments in neurocognitive functions and social cognition in SCZ [9, 10]. Under physiological conditions, females appear to be less susceptible to oxidative stress [29, 30]. This may be because estrogen can exert an antioxidant effect and play a protective role in cognitive function in SCZ [51, 52]. Thus, we speculate that lower levels of bilirubin in male SCZ patients with cognitive impairment may be the result of a relative lack of antioxidants or oxidative stress-induced bilirubin consumption. Unfortunately, estrogen was not investigated in this study. In future clinical studies, it is necessary to simultaneously identify the relationship between estrogen, bilirubin and cognitive function. Moreover, future research is needed to elucidate the possible molecular mechanisms that could underlie sex differences, such as whether estrogen improves cognition by affecting bilirubin metabolism pathways.

Some limitations of this study need consideration. First, various types of atypical antipsychotics were used to treat patients, which could be a confounder in interpreting our findings. We did not include types of atypical antipsychotics as a covariate because of a relatively small sample size in each sex; however, we added drug exposure as a covariate. Second, some confounding factors were not considered, such as refractoriness and estrogen level, because these data were not investigated. Third, some other prominent endogenous antioxidant cytoprotectants, such as GSH, were not investigated in this study. Fourth, this study was a cross-sectional study and could not determine the causality between decreased serum TBIL levels and cognitive deficits in patients with SCZ. Future longitudinal studies are needed to clarify their causality. Last but the most important limitation is that only a small magnitude of association is found between serum TBIL level and cognition in male patients. Thus, additional studies with a larger sample size and a better control on different types of atypical antipsychotics are warranted.

## Conclusion

Despite the aforementioned limitations, our study provides further support that dysregulation of serum TBIL levels could be associated with cognitive impairment in SCZ patients, though the magnitude of association is small. Moreover, for the first time, we report and address the sex difference in the relationship between serum TBIL levels and cognitive impairment in SCZ patients. It is very interesting to consider that novel therapeutic strategies aimed at achieving mild-to-moderate elevations of serum bilirubin (e.g., use of heme oxygenase-1) might be used in the future to improve cognition in male SCZ patients.

## Data Availability

The datasets used and/or analysed during the current study are available from the corresponding author on reasonable request.

## Abbreviations

SCZ: Schizophrenia
TBIL: Total bilirubin
PSM: Propensity score matching
MCI: Mild cognitive impairment
SIVD: Subcortical ischemic vascular disease
SALT-C: Schizophrenia by atypical antipsychotic treatment in China
DSM-IV: Diagnostic and statistical manual of mental disorders, fourth edition
DM: Diabetes mellitus
MoCA: Montreal cognitive assessment
TC: Total cholesterol
TG: Triglyceride
LDL: Low density lipoprotein
HDL: High density lipoprotein
OR: Odds ratios
CI: Confidence interval
VIF: Variance inflation factor
PSM: Propensity score matching
GSH: Glutathione
